# Selective Inhibition of ADAM17 Efficiently Mediates Glycoprotein Ibα Retention During Ex Vivo Generation of Human Induced Pluripotent Stem Cell‐Derived Platelets

**DOI:** 10.5966/sctm.2016-0104

**Published:** 2016-10-05

**Authors:** Shinji Hirata, Takahiko Murata, Daisuke Suzuki, Sou Nakamura, Ryoko Jono‐Ohnishi, Hidenori Hirose, Akira Sawaguchi, Satoshi Nishimura, Naoshi Sugimoto, Koji Eto

**Affiliations:** ^1^ Department of Clinical Application, Center for iPS Cell Research and Application (CiRA), Kyoto University, Kyoto, Japan; ^2^ Kaken Pharmaceutical Co., Ltd., Tokyo, Japan; ^3^ Kyoto Development Center, Megakaryon Co., Ltd., Kyoto, Japan; ^4^ Department of Anatomy, Faculty of Medicine, University of Miyazaki, Miyazaki, Japan; ^5^ Center for Molecular Medicine, Jichi Medical University, Tochigi, Japan; ^6^ Department of Innovation Stem Cell Therapy, Chiba University Graduate School of Medicine, Chiba, Japan

**Keywords:** Thrombopoiesis, Megakaryocyte, Induced pluripotent stem cell, Cell culture, Cell transplantation, Mitogen‐activated protein kinase

## Abstract

Donor‐independent platelet concentrates for transfusion can be produced in vitro from induced pluripotent stem cells (iPSCs). However, culture at 37°C induces ectodomain shedding on platelets of glycoprotein Ibα (GPIbα), the von Willebrand factor receptor critical for adhesive function and platelet lifetime in vivo, through temperature‐dependent activation of a disintegrin and metalloproteinase 17 (ADAM17). The shedding can be suppressed by using inhibitors of panmetalloproteinases and possibly of the upstream regulator p38 mitogen‐activated protein kinase (p38 MAPK), but residues of these inhibitors in the final platelet products may be accompanied by harmful risks that prevent clinical application. Here, we optimized the culture conditions for generating human iPSC‐derived GPIbα^+^ platelets, focusing on culture temperature and additives, by comparing a new and safe selective ADAM17 inhibitor, KP‐457, with previous inhibitors. Because cultivation at 24°C (at which conventional platelet concentrates are stored) markedly diminished the yield of platelets with high expression of platelet receptors, 37°C was requisite for normal platelet production from iPSCs. KP‐457 blocked GPIbα shedding from iPSC platelets at a lower half‐maximal inhibitory concentration than panmetalloproteinase inhibitor GM‐6001, whereas p38 MAPK inhibitors did not. iPSC platelets generated in the presence of KP‐457 exhibited improved GPIbα‐dependent aggregation not inferior to human fresh platelets. A thrombus formation model using immunodeficient mice after platelet transfusion revealed that iPSC platelets generated with KP‐457 exerted better hemostatic function in vivo. Our findings suggest that KP‐457, unlike GM‐6001 or p38 MAPK inhibitors, effectively enhances the production of functional human iPSC‐derived platelets at 37°C, which is an important step toward their clinical application. Stem Cells Translational Medicine *2017;6:720–730*

**Significance Statement**

This study is a tip for overcoming a practical but critical barrier for manufacturing human induced pluripotent stem cell (iPSC) platelets for clinical use. Unavoidable cleavage of glycoprotein Ibα during manufacturing at 37°C was completely blocked using KP‐457, a safe and selective ADAM 17 (a disintegrin and metalloproteinase 17 inhibitor), which enabled the platelets to exert improved hemostatic function in vivo. The strategy contributes to the realization of the clinical application of iPSC‐derived platelets.

## Introduction

Platelet transfusion is essential for the treatment of thrombocytopenia associated with hematologic diseases, trauma, and cancer chemotherapy. However, platelet concentrates have a short shelf life and, at present, can be supplied only from blood donations, which have associated risks of bacterial and viral infection. In addition, there are unmet needs for platelets expressing specific human leukocyte antigens or human platelet antigens. Human induced pluripotent stem cells (iPSCs) [1], which are available from diverse donors, could represent a potent new source of platelets that solves these problems. For this reason, we and others have recently developed an in vitro culture method whereby platelet‐producing megakaryocytes (MKs) are generated from human embryonic stem cells (ESCs) or iPSCs [2, 3, 4, 5, 6]. However, because one‐shot platelet transfusion requires more than 2–3 × 10^11^ platelets in a 200‐ml package, improvements in the efficiency of ex vivo platelet manufacturing are needed.

Donated platelets are stored at room temperature (20–24°C) to avoid a gradual loss of quality (i.e., platelet storage lesion) [7]. Stored platelets are subject to losing the extracellular domain of glycoprotein Ibα (GPIbα), which is enzymatically cleaved by a disintegrin and metalloproteinase 17 (ADAM17) (tumor necrosis factor [TNF]‐α converting enzyme) [8]. GPIbα is the receptor subunit of the GPIb/GPIX/GPV complex, which binds to von Willebrand factor (vWF) and enables platelets to attach to injured blood vessels under high shear stress. Without intact GPIbα, platelets lose the ability to properly attach to the vessel wall and thus become dysfunctional [8, 9, 10]. In addition, platelets without GPIbα are also rapidly cleared after transfusion [9, 10], indicating that high GPIbα expression is a critical factor to determine the lifetime of circulating platelets in vivo.

We previously demonstrated that the functionality of GPIbα^+^ iPSC‐derived platelets is intact and able to circulate and contribute to hemostasis in vivo [5], but GPIbα shedding also occurs during in vitro platelet generation from mouse or human ESCs/iPSCs [4, 5, 10]. We therefore examined different approaches to inhibit this phenomenon. Because donated platelets are stored at 24°C to inhibit GPIbα shedding, we assessed the effect of reducing the cultivation temperature used during the generation of iPSCs‐derived cells to 24°C. In addition, we examined the effect of pharmacologically inhibiting ADAM17 in cultures at 37°C. Panmetalloproteinase inhibitors with a hydroxamate structure, such as GM‐6001, have been used to inhibit GPIbα shedding during the storage of donated platelets and cultivated iPSC platelets [5, 9, 10]. In addition, p38 mitogen‐activated protein kinase (p38 MAPK) is activated during the storage of human and mouse platelets, and pharmacologic inhibition of p38 MAPK enhanced GPIbα retention, potentially through suppression of ADAM17 activation [11]. However, it is unknown whether p38 MAPK inhibition is effective at preventing GPIbα shedding from human iPSC‐derived platelets. In addition to these molecules, it was reported that metalloproteinase 8 (MMP8) inhibition during human iPSC platelet generation may prevent platelets from shedding GPIbα [2], although the contribution to shedding made by MMP8 has not been fully clarified. It thus remains unknown which molecule or combination of them is most effective at maximizing GPIbα retention during the generation of human iPSC platelets. In addition, to consider the final platelet product from culture media, safety concerns associated with components in the platelet transfusion package should be identified. Unfortunately, the safety of GM‐6001 and MMP8 inhibitor I (M8I) has never been determined, which may be an obstacle to the realization of clinical application because of potential risks of unexpected adverse health events in clinical use. In the present study, we show that the selective inhibition of ADAM17 using a novel selective and safe ADAM17 inhibitor, KP‐457, but not p38 MAPK, is effective for generating functional platelets from iPSCs.

## Materials and Methods

### Cells and Reagents

All reagents were obtained from Sigma‐Aldrich (St Louis, MO, http://www.sigmaaldrich.com) unless indicated otherwise. We used TKDA3‐4, a human iPSC clone; KhES‐3, a human ESC clone (a gift from Dr. H. Suemori, Institute for Frontier Medical Sciences, Kyoto University, Kyoto, Japan); and an immortalized megakaryocyte progenitor cell line (imMKCL, clone 7), which was previously established from iPSC‐derived hematopoietic progenitor cells (HPCs) [5, 12]. The mouse C3H10T1/2 feeder cell line was from the Riken BioResource Center (Tsukuba, Ibaraki, Japan, http://en.brc.riken.jp) [4, 5, 13]. Control human platelets were prepared from blood collected after obtaining signed informed consent from the donors and approval from the Ethical Committee of the Institute of Medical Science at the University of Tokyo (approval no. 22‐17‐0804). All studies involving the use of human samples were conducted in accordance with the Declaration of Helsinki.

The following antibodies were obtained from BD (Franklin Lakes, NJ, http://www.bd.com) unless indicated otherwise: allophycocyanin (APC)‐conjugated anti‐CD41a (integrin αIIb/β3 complex), eFluor450‐conjugated anti‐CD42a (GPIX) (eBioscience, San Diego, CA, http://www.ebioscience.com), phycoerythrin‐conjugated anti‐CD42b (GPIbα), V450, and APC‐conjugated anti‐CD9. A novel selective ADAM17 inhibitor with a reverse‐hydroxamate structure, KP‐457 [*N*‐(2‐{[4‐(but‐2‐yn‐1‐yloxy)phenyl]sulfonyl}‐1‐[4‐(methylsulfonamidomethyl)phenyl]ethyl)‐*N*‐hydroxyformamide], was supplied by Kaken Pharmaceutical Co., Ltd. (Tokyo, Japan). The panmetalloproteinase inhibitor GM‐6001, p38 MAPK inhibitor SB203580, MAPK/extracellular signal‐regulated kinase kinase (MEK) inhibitor U0126, IκB kinase β (IKKβ) inhibitor BMS345541, caspase inhibitor Z‐VAD, and protein kinase C (PKC) inhibitor Ro31‐8220 were from EMD Millipore (Billerica, MA, http://www.emdmillipore.com). The p38 MAPK inhibitor BIRB796 was from Axon Medchem (Groningen, Netherlands, https://www.axonmedchem.com). Human plasma was prepared by centrifugation from whole blood containing 10% acid citrate dextran, after which the supernatant was filtered through a 0.22‐µm filter (EMD Millipore).

### Hematopoietic Differentiation of Human iPSCs and ESCs

Directed differentiation to MKs and platelets from iPSCs and ESCs was performed as described previously [5, 13]. Briefly, 3 × 10^4^ HPCs derived from iPS‐sacs on C3H10T1/2 feeder cells in the presence of 20 ng/ml vascular endothelial growth factor were transferred on day 14 of culture onto C3H10T1/2 feeder cells in differentiation medium supplemented with 50 ng/ml stem cell factor, 100 ng/ml thrombopoietin, 25 U/ml heparin sodium, and test compounds at 24°C or 37°C. The medium was refreshed every 3 days, and nonadherent cells were collected and analyzed on days 22–24.

### Expansion of imMKCLs and Platelet Production

imMKCL expansion and subsequent platelet production were performed as previously reported [12]. Briefly, imMKCLs were cultured in the presence of doxycycline for self‐replication by the expression of three doxycycline‐regulated transgenes: *c‐MYC*, *BMI1*, and *BCL‐XL*. imMKCLs were subsequently cultured for 5 days in the absence of doxycycline to produce platelets by turning the transgenes off, with or without 15 µmol/l KP‐457.

### Flow Cytometric Analysis of In Vitro Differentiated MKs and Platelets

Flow cytometric analysis was performed as previously described [4, 5, 12, 13]. Briefly, iPSC‐ and ESC‐derived MKs on days 22–24 and platelets in their supernatants were stained with combinations of antibodies for 30 minutes on ice (for MKs) or at room temperature (for platelets). Samples of MKs/platelets were then washed, diluted with staining medium (phosphate‐buffered saline containing 3% fetal bovine serum), and analyzed using flow cytometry (FACSAria; BD). Trucount tubes (BD) were used to quantify the MKs and platelets.

### Carbonyl Cyanide *m*‐Chlorophenylhydrazone‐Induced GPIbα Shedding From Washed Platelets

Washed platelets were prepared as described elsewhere [5]. Platelets suspended in Tyrode‐HEPES buffer (1.5–2.5 × 10^8^ cells/ml) were treated with 100 µmol/l carbonyl cyanide *m*‐chlorophenylhydrazone (CCCP) at 37°C overnight in the absence or presence of inhibitors before further analysis.

### Flow Cytometry‐Based Platelet Aggregation Assay

Flow cytometry platelet aggregation assays were performed as recently described [12, 14]. Aliquots of iPSC platelets, imMKCL platelets, or fresh washed human platelets were stained with anti‐CD9‐APC or anti‐CD9‐V450, and CD41a^+^ platelets were counted using flow cytometry. Next, aliquots of platelets stained using anti‐CD9‐APC were mixed with platelets stained using anti‐CD9‐V450 at a 1:1 ratio (1–4 ×10^5^ cells/ml each) and suspended in Tyrode‐HEPES solution containing 20 mmol/l PPACK (a thrombin inhibitor), 10% filtered human plasma, and 3 mmol/l CaCl_2_. The mixed platelets were stimulated with 2 mg/ml ristocetin or 100 µmol/l TRAP6 and 50 µmol/l ADP for 10 minutes at 37°C while swirling, fixed, and analyzed using flow cytometry. To quantify platelet aggregation, an index of double‐colored events (%) was calculated using the following formula described elsewhere [14]:

Double‐colored events(%) =[APC^+^V450^+^/(APC^+^V450^+^+APC^+^V450^−^+APC^−^V450^+^)]×100

### MMP and ADAM Activity Assays

KP‐457 and GM‐6001 were tested for their ability to inhibit MMP‐ and ADAM‐catalyzed cleavage of substrates in a fluorescence‐based assay. Human ADAM17, ADAM10, and MMP17 were from R&D Systems (Minneapolis, MN, http://www.rndsystems.com), and human MMP1, MMP2, MMP3, MMP8, MMP9, MMP13, and MMP14 were from EMD Millipore. MMP1, MMP2, MMP8, MMP9, MMP13, and MMP17 were activated using *p*‐aminophenylmercuric acetate (Sigma‐Aldrich) before testing. Fluorogenic substrates for measuring activity of ADAM10 and ADAM17 [Nma‐LAQAVRSSK(Dnp)r‐NH_2_, based on the cleavage site of TNF‐α]; MMP1, MMP9, MMP13, and MMP14 [Dnp‐P‐Cha‐GC(Me)HAK(N‐Me‐Abz)‐NH_2_]; MMP3 [MOCAc‐RPKPVE‐Nva‐WRK(Dnp)‐NH_2_]; and MMP2, MMP8, and MMP17 [MOCAc‐PLGL‐A_2_pr(Dnp)‐AR‐NH_2_] were all provided by Peptide Institute (Osaka, Japan, https://www.peptide.co.jp/en) and used as substrates. In addition, inhibitory activities were measured using LC/MS/MS with a GPIbα‐based substrate peptide (KKTIPELDQPPKLRGVLQGHLESSRNDPFLHPDF), a C terminal‐based standard peptide (VLQGHLESSRNDPFLHPDF), and an internal standard peptide (VTTGKGQDHSPFWGF). These peptides were synthetized by Scrum (Tokyo, Japan, http://www.scrum‐net.co.jp/english/top_en.htm).

### Transmission Electron Microscopy Analysis

Transmission electron microscopy (TEM) was performed as described previously [12]. Samples were observed using a transmission electron microscope operating at 80 kV (HT‐7700; Hitachi, Tokyo, Japan, http://www.hitachi.com).

### Immunostaining and Confocal Microscopy Analysis

Confocal microscopy was performed as described previously [12]. All observations were made using an A1 confocal microscopic system (Nikon, Tokyo, Japan, http://www.nikon.com) equipped with an ×63/1.40 numerical aperture oil‐immersion objective. imMKCL‐derived platelets were attached on fibrinogen‐coated cover glass. For activation, platelets were treated with 200 µmol/l ADP and 40 µmol/l TRAP6. The cells were fixed, permeabilized, and stained with anti‐β1‐tubulin antibody (1:1,000; Medical & Biological Laboratories, Nagoya, Japan, http://www.mbl.co.jp), for detecting microtubule coils in resting platelets, or with rhodamine‐phalloidin (1:500; Thermo Fisher Scientific Life Sciences, Waltham, MA, http://www.thermofisher.com) and anti‐CD41a antibody (1:100; eBioscience), for detecting spreading F‐actin fibers in activated platelets. Alexa Fluor 488‐conjugated secondary antibody (1:500; Thermo Fisher) was also used.

### Clot Retraction Assay

Clot retraction assays were carried out as previously reported [12]. In brief, imMKCL platelets suspended in 20% platelet‐depleted plasma containing Iscove’s modified Dulbecco’s medium (3.6 × 10^8^ cells/ml) in the presence of thrombin (2 U/ml) were incubated at 37°C for 2 hours to induce clot formation and retraction.

### In Vivo Imaging for Thrombus Formation by iPSC platelets

The procedure was identical to that in the previous report [5, 12]. Small vessels (<25 μm in diameter) within mesenteric capillaries of male NOD/SCID/IL‐2Rg‐null (NOG) mice were evaluated. NOG mice were purchased from the Central Institute for Experimental Animals (Kawasaki, Kanagawa, Japan, http://www.ciea.or.jp/en/index.html).

### Statistical Analysis

Data are presented as means ± SEM. The statistical significance of observed differences was determined using Student’s two‐tailed *t* test for pairwise comparisons or Dunnett’s test for multiple comparisons. Significance of cell numbers was tested after logarithmic transformation. Values of *p* < .05 were considered significant.

## Results

### Culture at 37°C Is Critical for Efficient Generation of MKs and Platelets Exhibiting Good GP Retention

**Figure 1:**
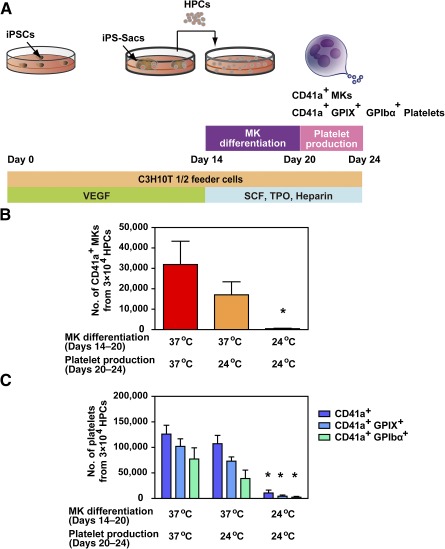
Cultivation at 37°C was necessary for efficient megakaryopoiesis and thrombopoiesis of human iPSC‐derived HPCs. **(A):** Schematic diagram of the in vitro differentiation protocol. To generate CD41a^+^ MKs and CD41a^+^ GPIX (CD42a)^+^ GPIbα (CD42b)^+^ platelets, human iPS‐sac‐derived HPCs were incubated with SCF, TPO, and heparin on C3H10T1/2 feeder cells at 24°C or 37°C during the MK differentiation phase (days 14–20) and platelet production phase (days 20–24). The numbers of MKs **(B)** and platelets **(C)** derived from iPSCs were measured under the different temperature conditions. ∗, *p* < .05 vs. 37°C culture condition on days 14–24 by Dunnett’s test, *n* ≥ 3. Abbreviations: GP, glycoprotein; HPC, hematopoietic progenitor cell; iPS, induced pluripotent stem; iPSC, induced pluripotent stem cell; MK, megakaryocyte; SCF, stem cell factor; TPO, thrombopoietin, VEGF, vascular endothelial growth factor.

To remain functional, ex vivo platelets must be maintained within a strict temperature window of 20–24°C. By contrast, it is commonly thought that iPSCs must be cultured at 37°C, but at that temperature, platelets undergo GPIbα shedding, perhaps as a result of metalloproteinase activation [10]. To assess the effect of reducing the ambient temperature to 24°C on iPSC platelet generation, we compared platelet yields and GPIIb (CD41a)/IIIa, GPIX (CD42a), and GPIbα (CD42b) levels after incubating the cells at 24°C or 37°C during the MK differentiation phase (days 14–20) or the platelet production phase (days 20–24) (Fig. 1A). Incubation at 24°C during days 14–24 resulted in the reduced yield of CD41a^+^ MKs from iPSC HPCs (Fig. 1B). Consistent with the reduction of MKs, platelet biogenesis based on the CD41a^+^, CD41a^+^GPIX^+^, and CD41a^+^GPIbα^+^ phenotypes was also diminished at 24°C (Fig. 1C). Likewise, the levels of individual glycoproteins were decreased (supplemental online Fig. 1), and the numbers of CD41a^+^GPIbα^+^ platelets were much lower than those of CD41a^+^GPIX^+^ platelets, even at 24°C (Fig. 1C). This fact is illustrated by the increase of platelets with the GPIbα^low^GPIX^high^ phenotype, which may be associated with the changes in cell metabolism or the shedding of GPIbα at 24°C (supplemental online Fig. 1A). These results demonstrate that cultivation at 37°C is requisite for normal platelet production.

### Inhibition of ADAM17 Using KP‐457 Is Sufficient to Retain GPIbα in CCCP‐Treated Aged Human Platelets

**Figure 2:**
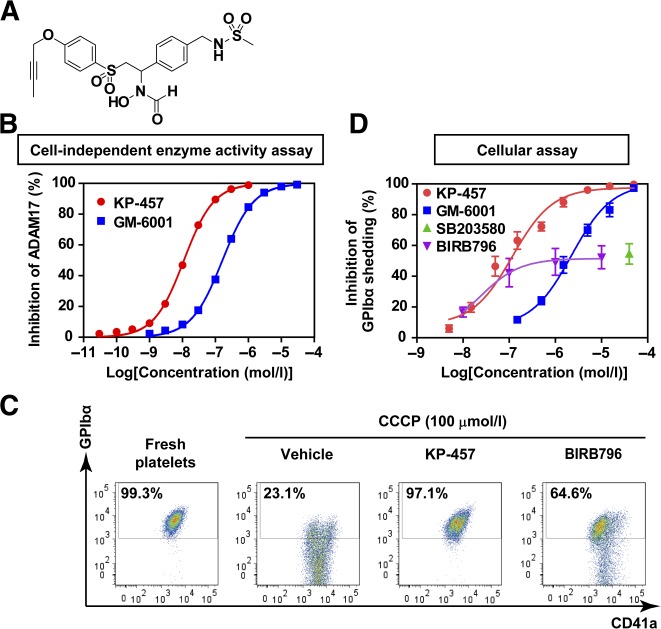
CCCP‐induced GPIbα shedding was completely blocked by the ADAM17 inhibitor KP‐457 but not by p38 MAPK inhibitors. **(A):** Structure of KP‐457. **(B):** Inhibitory effects of KP‐457 and GM‐6001 on ADAM17 enzymatic activity under cell‐free conditions. *n* = 3. **(C):** Representative FACS dot plots showing the inhibitory effect of KP‐457 (15 µmol/l) and BIRB796 (10 µmol/l) on CCCP‐induced GPIbα shedding from human washed platelets. Washed platelets were incubated with 100 µmol/l CCCP in the presence of inhibitors for 24 hours at 37°C. The GPIbα^+^ fraction among CD41a^+^ platelets was measured. **(D):** Dose‐dependent effects of the indicated inhibitors on GPIbα retention by platelets treated with CCCP. *n* ≥ 3. Abbreviations: ADAM, a disintegrin and metalloproteinase; CCCP, carbonyl cyanide *m*‐chlorophenylhydrazone; FACS, fluorescence‐activated cell sorting; GP, glycoprotein; MAPK, mitogen‐activated protein kinase.

Nonspecific metalloproteinase inhibitors [9] and p38 MAPK inhibitors [11] are both known to inhibit GPIbα shedding from human platelets. In iPSC platelets, the metalloproteinase inhibitor GM‐6001 and MMP8 inhibitor M8I both reportedly inhibit GPIbα shedding [2, 5, 10]. Unfortunately, M8I is not specific for MMP8 [15]. Like GM‐6001, M8I acts as a pan‐MMP/hydroxamate‐based inhibitor and thus potently inhibits ADAM17 [16]. We therefore developed a novel and selective ADAM17 inhibitor, KP‐457, that has a reverse‐hydroxamate structure and assessed its effects (Fig. 2A). ADAM17, also known as TNF‐α‐converting enzyme, cleaves various molecules such as GPIbα, GPV, and TNF‐α [17]. In cell‐free enzyme assays, KP‐457 inhibited cleavages of the TNF‐α sequence with 10 times the potency of GM‐6001 (Fig. 2B) and was >50 times more selective for ADAM17 than for other MMPs and ADAM10 (Table 1). In addition, we confirmed that the inhibition of C‐terminal cleavage of the GPIbα sequence by ADAM17 was concentration dependent, with a half‐maximal inhibitory concentration (IC_50_) of 10.6 nmol/l, whereas the IC_50_ for GM‐6001 was 53.8 nmol/l (supplemental online Fig. 2). KP‐457 at the lower concentration blocks Zn^2+^ chelation of the catalytic domain of ADAM17. Good laboratory practice studies confirmed that KP‐457 exhibited neither genotoxicity nor systemic toxicity at doses up to 3 mg/kg administered to dogs intravenously once a day for 4 weeks, which was the maximal dose tested (data not shown).

**Table 1:** Inhibitory profile of the novel selective ADAM17 inhibitor KP‐457

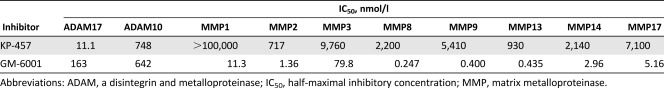

CCCP induces the cleavage of GPIbα in association with mitochondrial injury, and this effect can be blocked using GM‐6001 or a p38 MAPK inhibitor [9, 11]. Likewise, KP‐457 sustained intact GPIbα at levels seen in platelets freshly isolated from human blood (Fig. 2C, 2D). As in the cell‐independent (cell‐free) enzyme activity assays, KP‐457 inhibited GPIbα shedding with a potency 10 times that of GM‐6001 in the cellular assay (Fig. 2D), which is consistent with its aforementioned selective inhibition of ADAM17 activity (Fig. 2B; Table 1). These findings suggest that ADAM17 dominantly controls CCCP‐induced GPIbα shedding from human platelets. On the other hand, the inhibitory effects of the p38 MAPK inhibitors SB203580 and BIRB796 plateaued at a level around half the maximum inhibition elicited by KP‐457 or GM‐6001, which implies that ADAM17 is activated in part via a mediator other than p38 MAPK. We also confirmed that KP‐457 has no adverse effects on platelet aggregation induced by thrombin or collagen, whereas SB203580 and BIRB796 both diminished collagen‐induced platelet aggregation (supplemental online Fig. 3), as reported elsewhere [18].

### KP‐457, but not p38 MAPK Inhibitors, Retains the Expression of GPIbα on iPSC‐Derived Platelets

We next examined the ability of KP‐457 to inhibit GPIbα shedding during the generation of iPSC platelets in vitro. KP‐457 (15 μmol/l), GM‐6001 (50 μmol/l), or a p38 MAP kinase inhibitor (10 μmol/l) was administered during the MK differentiation and platelet production phases of HPCs cultivated at 37°C.

**Figure 3:**
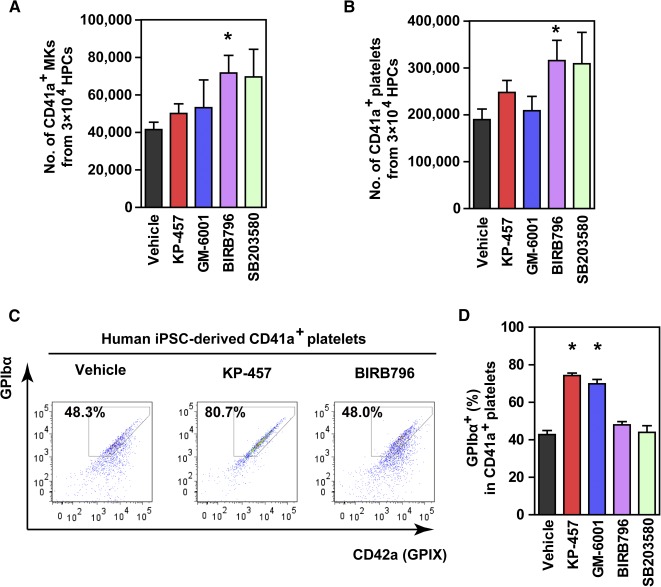
GPIbα shedding from platelets generated in vitro from iPSC‐MKs was effectively suppressed by KP‐457 but not by p38 MAPK inhibitors. **(A)** Numbers of CD41a^+^ iPSC‐derived MKs. Human HPCs were incubated for 8–10 days in differentiation medium supplemented with stem cell factor, thrombopoietin, heparin, and/or inhibitors at 37°C, followed by flow cytometric analysis. p38 MAPK inhibitors increased MK yields, whereas KP‐457 and GM‐6001 had only minor effects. **(B)** Numbers of CD41a^+^ iPSC platelets. **(C)** Representative FACS dot plots of platelets generated from iPSC‐derived HPCs in the presence of KP‐457 or BIRB796. **(D)** Percentages of the GPIbα^+^ population in CD41a^+^ platelets. Metalloproteinase inhibitors, including KP‐457, but not p38 MAPK inhibitors, enabled GPIbα retention by iPSC‐derived platelets. ∗, *p* < .05 vs. vehicle group by Dunnett’s test, *n* ≥ 4. Abbreviations: FACS, fluorescence‐activated cell sorting; GP, glycoprotein; HPC, hematopoietic progenitor cell; iPSC, induced pluripotent stem cell; MAPK, mitogen‐activated protein kinase; MK, megakaryocyte.

KP‐457 or GM‐6001 had no significant effect on the number of MKs (Fig. 3A) or CD41a^+^ total platelets (Fig. 3B). Importantly, these inhibitors were able to retain the expressions of GPIbα on CD41a^+^ platelets (Fig. 3C, 3D). GPIbα retention by KP‐457 was consistently observed in platelets derived from human ESCs (supplemental online Fig. 4A) and imMKCLs [12] (supplemental online Fig. 4B).

On the other hand, BIRB796 and SB203580 increased MK numbers (Fig. 3A). This increase may be attributable to an increased expansion of CD34^+^ HPCs related to the p38 MAPK inhibition [19]. As a result, the number of CD41a^+^ total platelets was increased (Fig. 3B). The inhibition of p38 MAPK apparently did not affect platelet release from MKs. Unfortunately, p38 MAPK inhibitors failed to preserve GPIbα in iPSC‐derived platelets (Fig. 3C, 3D). This result suggests that p38 MAPK inhibition promotes megakaryopoiesis but has no effect on GPIbα shedding from iPSC‐derived platelets.

**Figure 4:**
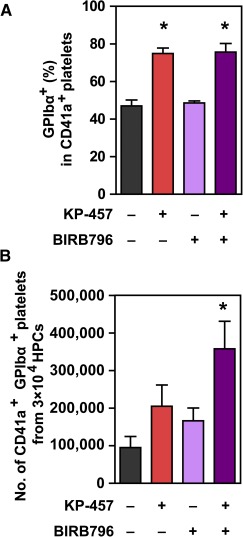
Combination of KP‐457 and BIRB796 exerted an additive effect on GPIbα^+^ platelets yields. Percentages of the GPIbα^+^ population in CD41a^+^ platelets **(A)** and numbers of CD41a^+^ GPIbα^+^ platelets generated from human iPSCs **(B)** with or without KP‐457 (15 µmol/l) and BIRB796 (10 µmol/l). ∗, *p* < .05 vs. vehicle group by Dunnett’s test, *n* = 4. Abbreviations: GP, glycoprotein; HPC, hematopoietic progenitor cell; iPSC, induced pluripotent stem cell.

Because both metalloproteinase inhibitors and p38 MAPK inhibitors increase the number of GPIbα^+^ iPSC platelets, the former by increasing the ratio of the GPIbα^+^ population and the latter by increasing the total number of platelets, we sought to evaluate whether the simultaneous addition of these inhibitors could further increase the number of GPIbα^+^ iPSC platelets. In combination, KP‐457 and BIRB796 exhibited an additive effect on the GPIbα^+^ platelet yield, but not on the percentage of the GPIbα^+^ population (Fig. 4), which is consistent with our observations that these two inhibitors contribute to an increase in GPIbα^+^ iPSC platelets through separate mechanisms of action.

### Direct Inhibition of ADAM17 Is the Most Effective Strategy for Retaining GPIbα During iPSC Platelet Generation

**Figure 5:**
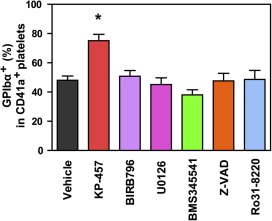
Direct inhibition of ADAM17 more effectively sustained GPIbα retention by cultured iPSC‐derived platelets than suppression of potential upstream activators of ADAM17. Percentages of the GPIbα^+^ population in CD41a^+^ iPSC‐derived platelets treated with the indicated inhibitors during the platelet production phase (days 21–23 or 24). KP‐457 (15 μmol/l), BIRB796 (10 μmol/l), MAPK/extracellular signal‐regulated kinase kinase inhibitor U0126 (10 μmol/l), IkB kinase β inhibitor BMS345541 (10 μmol/l), caspase inhibitor Z‐VAD (10 μmol/l) and protein kinase C inhibitor Ro31‐8220 (3 μmol/l) were used. Inhibition of potential ADAM17 activator molecules failed to prevent GPIbα shedding evoked by cultivation at 37°C. ∗, *p* < .05 versus vehicle group by Dunnett's test, *n* ≥ 3. Abbreviations: ADAM, a disintegrin and metalloproteinase; GP, glycoprotein; iPSC, induced pluripotent stem cell.

Although p38 MAPK is a putative upstream signaling molecule contributing to ADAM17 activation during platelet preservation [11], our results indicate it has no involvement in GPIbα shedding during iPSC platelet generation. To clarify the mechanism by which ADAM17 is activated during iPSC platelet generation, we tested the effects of inhibiting other putative upstream molecules, including MEK1/2, IKKβ, caspases, and PKCs [17, 20], all of which have been implicated in the activation of ADAM17. The respective inhibitors were administered for the final 2–3 days of culture, because some of them suppressed differentiation to MKs and thrombopoiesis (data not shown). None of the tested inhibitors other than KP‐457 prevented the loss of GPIbα (Fig. 5). We therefore concluded that selective and direct inhibition of ADAM17 is the most effective strategy for retaining GPIbα during iPSC platelet generation.

### GPIbα‐Retaining Platelets Generated Using KP‐457 Exhibited Improved Hemostatic Function In Vivo

**Figure 6:**
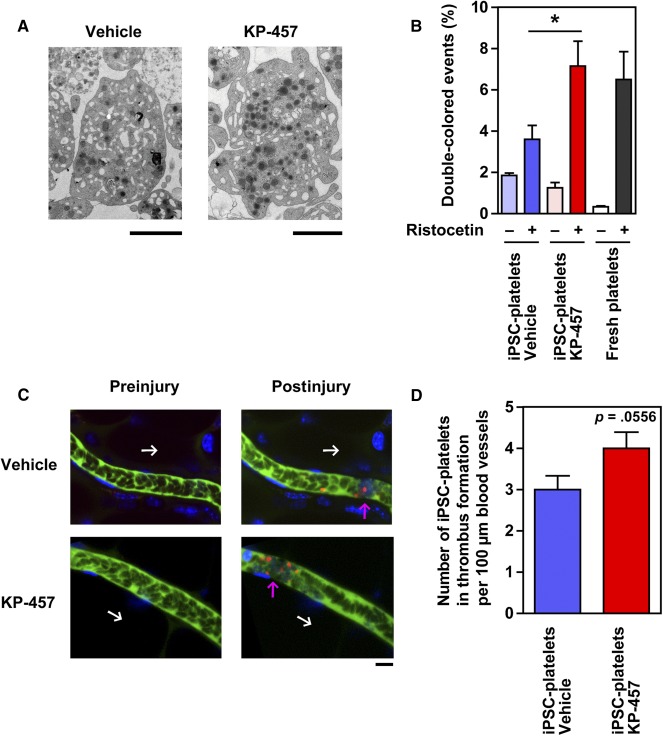
GPIbα‐retaining iPSC platelets generated in the presence of KP‐457 exhibited an improved response to vWF in vitro and hemostatic function in vivo. **(A):** Transmission electron micrographs of imMKCL platelets produced with or without KP‐457. Scale bar, 2 µm. **(B):** Percentage of double‐colored events served as an aggregation index in a flow cytometry‐based platelet aggregation assay using peripheral blood‐derived platelets or iPSC‐derived platelets generated with or without 15 µmol/l KP‐457. A mixture of APC‐ and V450‐labeled platelets was stimulated using 2 mg/ml ristocetin to evoke vWF/GPIbα‐dependent platelet aggregation. ∗, *p* < .05 vs. without KP‐457, *n* ≥ 4. **(C):** Representative sequential images of GPIbα^+^ iPSC platelets generated with KP‐457 in mouse thrombus formation model. A combination of FITC‐dextran (green), Hoechst33342 (blue), and equal numbers of tetramethylrhodamine‐labeled iPSC platelets (1 × 10^7^ CD41a^+^ platelets, red) generated with or without KP‐457 were transfused into irradiated NOG mice. Hematoporphyrin was also administered to induce thrombus formation by chemical reaction after laser‐induced injury. Mesenteric capillaries were visualized using a confocal laser scanning microscope. Attachment of platelets including tetramethylrhodamine‐positive iPSC platelets on injured blood vessels and subsequent thrombus formation were observed after the injury. White arrow, direction of blood flow; pink arrow, site of thrombus formation. Scale bar, 10 µm. **(D):** The number of iPSC platelets in thrombus formation was estimated using the images of 30 vessels from two mice of the same group in two independent experiments. GPIbα‐retained iPSC‐derived platelets with KP‐457 exerted more effective hemostatic function in vivo. ∗, *p* = .0556 vs. vehicle group, *n* = 30. Abbreviations: APC, allophycocyanin; GP, glycoprotein; imMKCL, immortalized megakaryocyte progenitor cell line; iPSC, induced pluripotent stem cell; vWF, von Willebrand factor.

We previously demonstrated that directly differentiated iPSC‐derived platelets or imMKCL‐derived platelets exhibit platelet‐like phenotypes, including granule contents inside the cell bodies, platelet agonist‐responsive activations, circulation in bloodstream, and contribution to hemostasis in vivo [5, 12]. Treatment with KP‐457 seemingly improved or enhanced the structural properties of normal platelets according to TEM (Fig. 6A), microtubule coil formation detected by β1‐tubulin staining (supplemental online Fig. 5A), actin reorganization via outside‐in signaling (supplemental online Fig. 5B), aggregation (supplemental online Fig. 5C, 5D), and clot formation (supplemental online Fig. 5E).

To further examine the functionality of the GPIbα‐retaining iPSC platelets generated in the presence of KP‐457 in vitro, we used a flow cytometry‐based platelet aggregation assay to assess vWF/GPIbα‐dependent platelet aggregation induced by ristocetin [12, 14]. Upon the stimulation of a mixture of equal densities of APC‐labeled and V450‐labeled platelets, a double‐positive population was increased, and single‐positive populations were conversely decreased, with platelet aggregation. We found that iPSC platelets generated in the presence of KP‐457 exhibited greater aggregation capability than those generated in its absence and that this capability resembled that of peripheral blood‐derived fresh platelets (Fig. 6B). Next, we examined overall hemostatic function in vivo by the iPSC platelets. The platelets were transfused into immunodeficient NOG mice with irradiation‐induced thrombocytopenia and monitored using an in vivo imaging system [5, 12, 21]. When equal numbers (1 × 10^7^ cells) of tetramethylrhodamine‐labeled CD41a^+^ iPSC platelets generated with or without KP‐457 were transfused into the mice before laser/reactive oxygen species‐induced vessel injury, a higher number of iPSC platelets contributed to thrombus formation in the KP‐457‐treated group than in platelets without KP‐457 (Fig. 6C, 6D; *p* = .0556). Based on these results, we concluded that KP‐457 successfully protects the GPIb/GPIX/GPV complex formation required for thrombus formation in vivo.

## Discussion

Surface GPIbα expression is a critical determinant of platelet half‐life in vivo and mediates the attachment of circulating platelets to damaged blood vessels under high shear stress [9, 10, 22]. The level of intact GPIbα on the platelet surface is controlled via ADAM17‐catalyzed shedding of the GPIbα ectodomain [8, 23, 24]. Avoiding ADAM17‐mediated shedding of GPIbα using a nonselective MMP inhibitor improved both posttransfusion kinetics and hemostatic function of mouse platelets derived from peripheral blood or mouse ESCs [8, 9, 10]. Here, we confirmed that selective ADAM17 inhibition using KP‐457 was similarly effective for human ESC‐ and iPSC‐derived platelets generated at 37°C, which otherwise underwent GPIbα shedding (Figs. 3–5; supplemental online Fig. 4). Moreover, the cultivation at 37°C was requisite for normal platelet production (Fig. 1; supplemental online Fig. 1). In contrast, platelets as well as MKs created at 24°C expressed decreased levels of glycoproteins (supplemental online Fig. 1), leading to insufficient hemostasis.

Although studies with ADAM17‐deficient mice have shown that GPIbα shedding from mouse platelets is ADAM17‐dependent [8, 11, 25], the dependency on ADAM17 had not been confirmed in human platelets. Such confirmation is important because the substrate selectivity of ADAM17 and ADAM10 can differ depending on species and the type of activating stimuli [17, 25, 26]. The administration of KP‐457, a selective ADAM17 inhibitor, had a protective effect on platelet GPIbα levels in platelets derived from both human peripheral blood and iPSCs that was well correlated with ADAM17 inhibition (Fig. 2 and 3). Consistent with the importance of ADAM17 inhibition, the human GPIbα sequence contains a putative ADAM17 cleavage site at V465, and ADAM17, but not ADAM10, cleaves the GPIbα‐based peptide [27]. On the other hand, M8I also effectively supports the production of functional iPSC‐derived platelets [2]. This mechanism does not appear to be attributable to pure MMP8 inhibition, as M8I is a panmetalloproteinase inhibitor and not selective for MMP8 [15], indicating it could potently inhibit ADAM17 [16]. We therefore concluded that GPIbα shedding from human platelets is dependent on the activity of ADAM17 but not the activity of ADAM10 or MMP8.

Our present findings indicate that KP‐457 strongly blocks GPIbα shedding during in vitro aging of platelets derived from peripheral blood, ESCs, and iPSCs (Figs. 2–5; supplemental online Fig. 4) without affecting other platelet phenotypes; e.g., formation of microtubule coils, outside‐in signaling, platelet aggregation, and clot formation (supplemental online Figs. 3, 5). Interestingly, KP‐457 administration improved platelet structure according to TEM (Fig. 6A). Moreover, upon ristocetin stimulation, iPSC platelets prepared in the presence of KP‐457 display an improved response to ristocetin that is not inferior to that of peripheral blood platelets (Fig. 6B). More important, iPSC platelets generated with KP‐457 successfully participated in hemostasis in vivo (Fig. 6C, 6D), which implies that the functionality of GPIb/IX/V complexes is sustained by KP‐457.

In contrast to KP‐457, p38 MAPK inhibitors did not protect GPIbα on iPSC platelets (Figs. 3–5). Oxidative stress induces p38 MAPK‐dependent digestion of GPIbα in human and mouse platelets [23], and pharmacologic inhibition of p38 MAPK blocks GPIbα shedding from platelets treated with H_2_O_2_, putatively via ADAM17 inhibition [11]. However, it has been suggested that p38 MAPK inhibition by SB203580 suppresses thrombin‐induced GPIbα shedding from mouse platelets less effectively than GM‐6001 [28]. This observation suggests that whether selective inhibition of p38 MAPK is sufficient for GPIbα retention may depend on the experimental conditions. We observed that the p38 MAPK inhibitors SB203580 and BIRB796 inhibited CCCP‐induced GPIbα shedding from human peripheral blood platelets to some extent (Fig. 2C, 2D), which is consistent with earlier reports [11].

Activation of ADAM17 is tightly regulated through its expression, phosphorylation, translocation, and localization on the cell membrane, and also through intramolecular disulfide bridge formation within its extracellular domains, which are mediated via several signaling pathways [17]. We sought to explore the signaling pathways leading to ADAM17 activation during iPSC platelet generation by blocking various intracellular mediators, with a primary focus on kinases, but unfortunately no dominant pathway was found (Fig. 5). Meanwhile, a study that used mouse ADAM17 without its cytoplasmic domain suggests the possibility that specific phosphorylation in the domain is not necessarily required for ADAM17 activation [29]. Also, iPSC platelets after release from the MKs could be continuously affected by the culture itself, i.e., changes in pH, temperature, and oxygen partial pressure, which might cause ADAM17 activation through unknown kinases or cytoplasmic domain‐independent mechanisms.

p38 MAPK inhibition had no positive effect on GPIbα maintenance in iPSC platelets (Figs. 3C, 3D, and 4A) but increased the total number of iPSC platelets by increasing the number of iPSC‐MKs (Fig. 3A, 3B), and combination with ADAM17 inhibition additively increased the yield of GPIbα^+^ platelets (Fig. 4B). Inhibition of p38 MAPK using inhibitors or short hairpin RNAs (shRNAs) reportedly affects several stages of hematopoiesis and megakaryopoiesis in vitro, including the expansion of cord blood‐derived CD34^+^ HSPCs [19], specification into the MK lineage, and maturation [30], which may account for the ability of p38 MAPK inhibitors to increase the number of iPSC‐MKs during HPC culture. Were a clinically safe p38 MAPK inhibitor to become available, its combined use with KP‐457 could improve the production of iPSC‐derived platelets.

The present study focuses on GPIbα shedding but does not address the shedding of GPVI, a collagen receptor that regulates platelet activation signaling [31]. Patients with GPVI defects or compound heterozygous mutations of GPVI display mild bleeding [31, 32, 33]. GPVI shedding is observed during platelet generation from mouse ESCs and is partially inhibited by the nonselective MMP inhibitor GM‐6001 at a high concentration (100 µmol/l) [10]. GPVI shedding is suspected during in vitro human platelet generation [12, 14]. In humans, ADAM10 is a major sheddase of it [27]. Because KP‐457 and GM‐6001 had similar IC_50_ values against ADAM10 (Table 1), comparable effects of KP‐457 on GPVI shedding would be expected. To further improve the function of the platelets generated in vitro, the retention of GPVI expression should be addressed in the next step.

For successful clinical application of iPSC technology to platelet transfusion, production of platelets in large quantity and high quality is needed. We recently established an imMKCL system for robust self‐replication and platelet production [12]. The imMKCL system requires 5‐day cultivation for platelet production, in which the platelets are released between days 3 and 5 (data not shown). In accordance with our observation that GPIbα expression on platelets is highly maintained for 3 days in the presence of KP‐457 under static culture conditions at 37°C (data not shown), we confirmed that KP‐457 consistently facilitated the generation of imMKCL platelets of higher quality based on GPIbα expression level (supplemental online Fig. 4B). Meanwhile, recent reports have revealed that a new platelet storage buffer, BRS‐A, which is based on clinically available bicarbonated Ringer’s solution (BRS) and acid‐citrate‐dextrose formula A, successfully maintains human platelets, including GPIbα expression, for 7 days at room temperature [34]. Combination of KP‐457 for the generation of GPIbα^+^ iPSC platelets at 37°C and BRS‐A solution for storage at room temperature (22–24°C) may contribute to a donor‐independent iPSC platelet supply system aimed at clinical use.

Also for the goal of clinical application, a GPIbα shedding inhibitor that is well tolerated should be chosen. Because platelet concentrates contain material residues when intravenously injected, the materials should be eliminated before injection if they are toxic. However, it is not easy to purify platelets completely without platelet activation because of platelets’ inherent fragility. Therefore, GM‐6001, which needs high concentrations for its action, may be unsuitable for clinical use. Additionally, nonselective metalloproteinase inhibitors were reported to induce musculoskeletal inflammation in clinical trials, potentially because of the broad inhibition of metalloproteinases [35]. The selective ADAM17 inhibitor KP‐457 was developed and confirmed to have very little risk of genetic or systemic toxicities. Overall, KP‐457 will be a favorable process aid for GPIbα retention on platelets generated in culture, which will improve platelet quality.

## Conclusion

Our findings indicate that the selective blockade of ADAM17 using KP‐457 safely enables iPSC‐derived platelets to retain GPIbα and thus remain functional. These effects are an important step toward the realization of platelet clinical applications.

## Author Contributions

S.H., T.M., and R.J.‐O.: conception and design, collection and/or assembly of data, data analysis and interpretation, manuscript writing; D.S., S. Nakamura, H.H., and A.S.: collection and/or assembly of data, data analysis and interpretation; S. Nishimura: collection and/or assembly of data, data analysis and interpretation, manuscript writing; N.S.: data analysis and interpretation, manuscript writing; K.E.: conception and design, collection and/or assembly of data, data analysis and interpretation, manuscript writing, final approval of manuscript.

## Disclosure of Potential Conflicts of Interest

This study was performed as a collaboration between K.E. and Kaken Pharmaceutical Co., Ltd. K.E. is founder of Megakaryon Co., Ltd. The interests of K.E. were reviewed and are managed by Kyoto University in accordance with its conflict‐of‐interest policies. K.E. had research funding from Kaken Pharmaceutical Co., Ltd., from 2010 to 2013. S.H., T.M., R.J‐O., and K.E. have applied for a patent related to this manuscript. S.H. and T.M. are employees of Kaken Pharmaceutical Co., Ltd. The other authors indicated no potential conflicts of interest.

## Supporting information

Supporting Information
