# Supplementary material for: Selective Inhibition of ADAM17 Efficiently Mediates Glycoprotein Ibα Retention During Ex Vivo Generation of Human Induced Pluripotent Stem Cell‐Derived Platelets
Source: Stem Cells Transl Med. 2016 Oct 5;6(3):720–30. doi: 10.5966/sctm.2016-0104 (PMC5442763; doi:10.5966/sctm.2016-0104)
Supplement: Supplementary file 1 — Supporting Information [file SCT3-6-0720-s001.pdf]

Supplemental Information - Eto et al.

Supplemental Figures

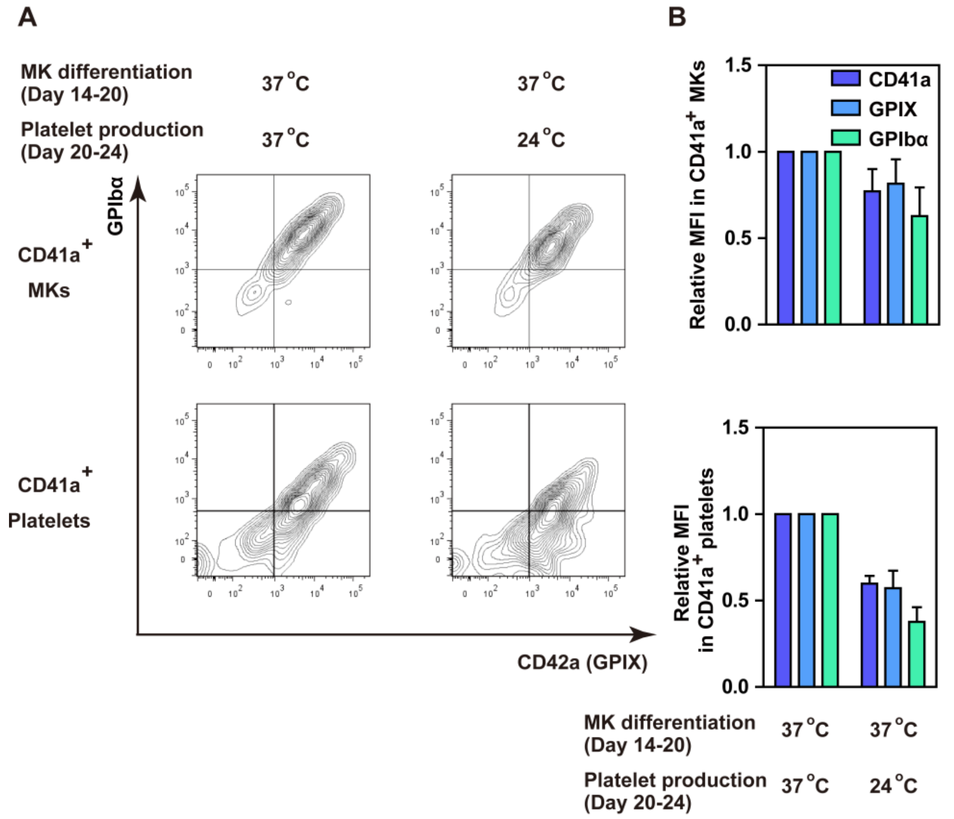

Fig. S1. Decreased expression of CD41a, GPIX and GPIbα on megakaryocytes and platelets generated at 24 °C during the platelet production phase.

(A) Representative FACS patterns of MKs and platelets differentiated from iPSC-derived HPCs under 37 °C or 24 °C culture conditions on day 24 (as shown in Fig. 1). (B) Relative expression of the indicated glycoproteins in CD41a$^+$ MKs and CD41a$^+$ whole platelets derived from iPSCs under the indicated temperature conditions. Levels of CD41a, GPIX and GPIbα were obviously lower on MKs and platelets generated at 24 °C during days 20-24 than those generated at 37 °C throughout the entire culture period. N=2.

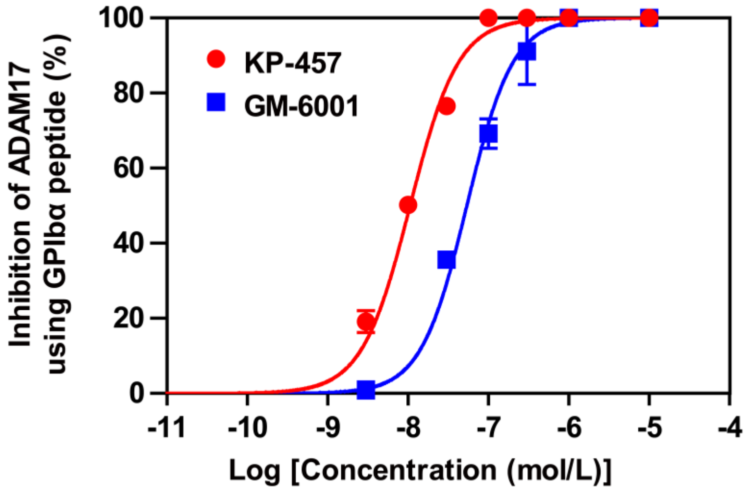

Fig. S2. KP-457 and GM-6001 dose-dependently blocked digestion of a human GPIbα peptide sequence by human ADAM17.

Inhibitory effects of KP-457 and GM-6001 on cleavage of a human GPIbα sequence by human ADAM17. KP-457 was a more potent ADAM17 antagonist than GM-6001. These results are similar to those in Fig. 2B. N=3.

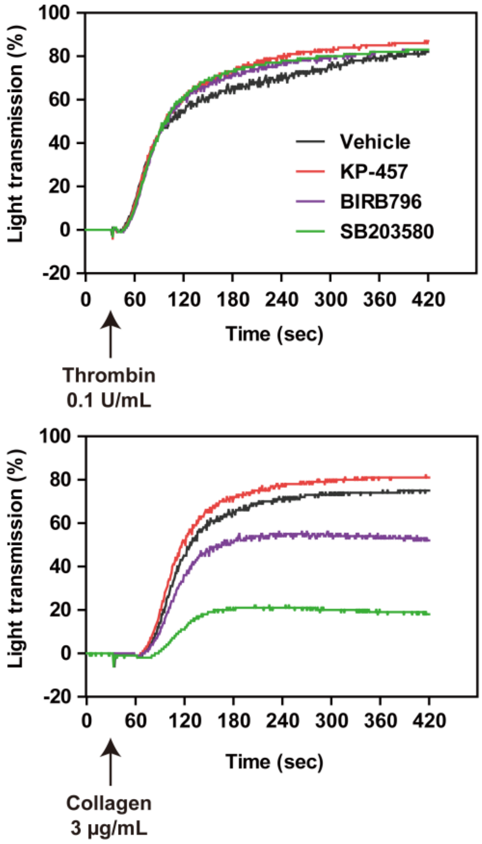

Fig. S3. No obvious effect of KP-457 on aggregation of human washed platelets.

Platelet aggregation was induced using 0.1 U/mL thrombin or 3 μg/mL collagen in the absence or presence of KP-457 (15 μmol/L), SB203580 (40 μmol/L) or BIRB796 (10 μmol/L).

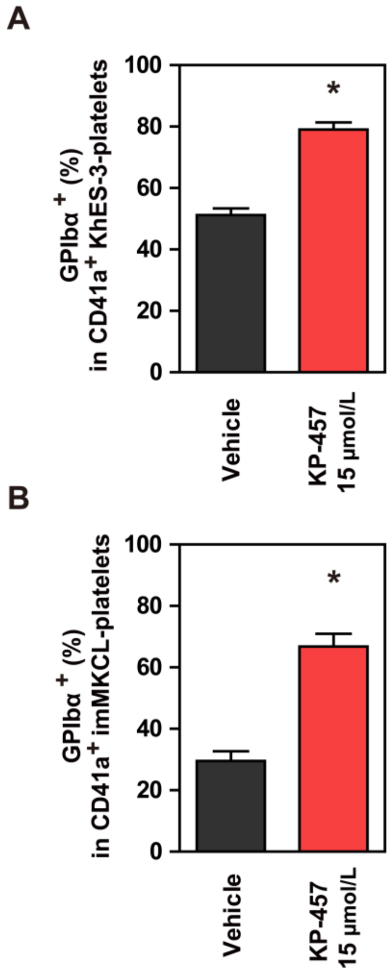

Fig. S4. The inhibitory effect of KP-457 on GPIbα shedding in platelets derived from human ESCs and immortalized megakaryocyte progenitor cell lines (imMKCLs).

Percentages of GPIbα-expressing $CD41a^+$ platelets produced from human ESCs (A) and immortalized megakaryocyte progenitor cell lines (imMKCLs) (B), in the absence or presence of KP-457 (15 μmol/L). * $P < 0.05$ compared to the vehicle group by student's *t* test, N≧3.

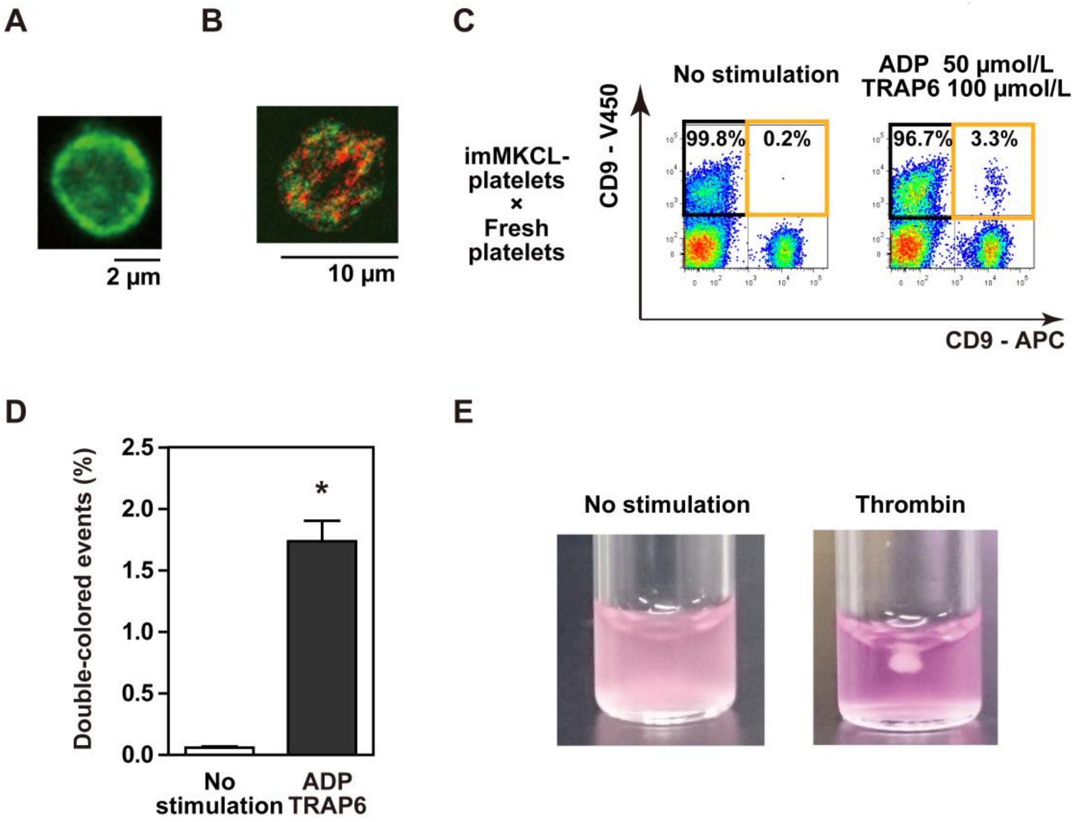

Fig. S5. Structural and functional characterization of imMKCL-platelets generated in the presence of KP-457.

Representative confocal microscopic images of resting platelets (A) or platelets after stimulation with ADP and TRAP6 (B). In (A), green indicates ⍰1 tubulin. Scale bar: 2 µm. In (B), green indicates CD41a and red indicates actin. Scale bar: 10 µm. (C) Flow cytometric detection of aggregated platelets as a double-positive population among human fresh platelets stained with CD9-APC and imMKCL-platelets stained with CD9 V450 after stimulation with ADP and TRAP6. (D) Percentage of double-colored events of stimulated platelets in C. * $P < 0.05$ compared to the no stimulation group by student's *t* test, N=3. (E) Clot retraction test: imMKCL-platelets (3.6×10^{8} platelets/mL) in 20% platelet-depleted human plasma containing Iscove's modified Dulbecco's medium were stimulated with thrombin (2 U/mL) for 2 hr.
